# Whole-Genome Survey and Microsatellite Marker Detection of Antarctic Crocodile Icefish, *Chionobathyscus dewitti*

**DOI:** 10.3390/ani12192598

**Published:** 2022-09-28

**Authors:** Jinmu Kim, Seung-Jae Lee, Euna Jo, Eunkyung Choi, Minjoo Cho, Soyun Choi, Jeong-Hoon Kim, Hyun Park

**Affiliations:** 1 Division of Biotechnology, College of Life Sciences and Biotechnology, Korea University, Seoul 02841, Korea; 2 Korea Polar Research Institute (KOPRI), Incheon 21990, Korea

**Keywords:** *Chionobathyscus dewitti*, crocodile icefish, Illumina Novaseq 6000, Jellyfish, GenomeScope, K-value, microsatellite

## Abstract

**Simple Summary:**

Crocodile icefish inhabit the deep sea around the Southern Ocean and belong to the family Channichthyidae. The species lacks hemoglobin and has evolved an antifreeze protein, unlike other teleosts. In this study, the whole-genome survey and microsatellite motifs were analyzed, which provide relevant information on genetic diversity, population genetics, and the genomic study of crocodile icefish.

**Abstract:**

The crocodile icefish, *Chionobathyscus dewitti*, belonging to the family Channichthyidae, is an endemic species of the Southern Ocean. The study of its biological features and genetics is challenging as the fish inhabits the deep sea around Antarctic waters. The icefish, the sole cryopelagic species, shows unique physiological and genetic features, unlike other teleosts. It lacks hemoglobin and has evolved antifreeze proteins. Here, we report the genome sequencing data of crocodile icefish produced using the Illumina Novaseq 6000 platform. The estimated genome size was 0.88 Gb with a K-value of 19, and the unique sequence, heterozygosity, error, and duplication rates were 57.4%, 0.421%, 0.317%, and 0.738%, respectively. A genome assembly of 880.69 Mb, with an N50 scaffold length of 2401 bp, was conducted. We identified 2,252,265 microsatellite motifs from the genome assembly data, and dinucleotide repeats (1,920,127; 85.25%) had the highest rate. We selected 84 primer pairs from the genome survey assembly and randomly selected 30 primer pairs for validation. As a result, 15 primer pairs were validated as microsatellite markers.

## 1. Introduction

Over the past 40 million years, the Antarctic shelf has been affected by a series of structural and oceanographic events that have changed the composition of fish fauna and initiated fauna substitution [1]. At the core of these various events, the Drake Passage between southern South America and the Antarctic Peninsula dates back 23.5 million to 32.5 million years, dividing notothenioid stock into Magellan (non-Antarctic) and Antarctic components [2]. Over time, water temperature drops, ice forms, and salinity changes in the Drake Passage [3], making it harder to capture specimens around the polar regions. Because the Antarctic Peninsula has seen the most severe local consequences of climate change in the polar regions [3], it is critical to determine whether Antarctic fish are experiencing any loss of genetic variety.

The variety of adaptations that evolved in Antarctic fish enabling their survival in freezing temperatures represents the extremes of cold adaptation in vertebrates [4]. The Antarctic sea, perennially at or near freezing, makes the oxygen concentration higher than that in other oceans [4]. Species belonging to the Channichthyidae (Teleosteri: Perciformes) family inhabit the Antarctic sea and are white-blooded icefish known for their unique physiological characteristics because of the absence of hemoglobin, an oxygen-binding protein in the blood [5]. Dissolved oxygen can be freely transported in solution, but most animals carry oxygen into the respiratory tissue by one or more respiratory proteins. Like other vertebrates, in fish, hemoglobin carries oxygen to cells [1]. Hemoglobin is highly temperature sensitive, and its structural and functional properties may in part reflect its environmental history, which may benefit many biological questions [1]. High-Antarctic notothenioids have evolved to reduce the hemoglobin concentration and multiplicity, and even erythrocyte numbers, compared with temperate and tropical species [6,7].

The crocodile icefish, *Chionobathyscus dewitti*, with various unique biological features, belongs to the family Channichthyidae and inhabits the Southern Ocean. It is known as one of the significant prey of the Antarctic toothfish, *Dissostichus mawsoni* [8]. *C. dewitti* occurs in the deep sea (up to 2000 m) around the Southern Ocean, but its exact habitat is unknown [9]. As a result, this species may be an excellent model for explaining the distribution and evolution of Antarctic fish. There have been no relevant genetic markers established for this species, nor has there been any research to understand the population genetic structure to investigate the evolution of this species.

These investigations necessitate the use of microsatellites, which have proven to be useful and reliable genetic markers in genetic studies [10,11]. In the past, the most popular method for isolating microsatellites was to identify repeat-containing sequences from libraries of genomic DNA enriched for microsatellite motives (Type II loci, [11,12]). Currently, these markers are isolated from sequencing libraries acquired by next-generation sequencing (NGS) techniques, which have proven to be more cost effective than the previous labor-intensive and costly approach [13]. Various pipelines are still being developed to select accurate microsatellite marker using the NGS technique, and we classified by in silico microsatellite detection and the primer design of crocodile icefish using QDD pipeline version 3.1.2 [14], and this information will help improve the conservation measures of crocodile icefish. 

*C. dewitti* genome analysis is required to identify the biological and genetic features that enable it to live in extremely cold environments. Using NGS, we estimated the genomic characteristics of *C. dewitti* and investigated the occurrence of microsatellite. These results will aid further whole-genome sequencing of *C. dewitti* and the development of new molecular markers for population genetics.

## 2. Materials and Methods

### 2.1. Sample Collection and Genome Sequencing

*C. dewitti* specimens (length: ~30 cm) were captured from the Southern Ocean (65°05′S, 170°30′ E on CCAMLR Subarea 88.1) in Antarctica. The samples were then transferred to a freezer and transported to the laboratory. Muscle tissue from a single female specimen was anatomized from the sample and isolated using the conventional phenol-chloroform method to extract pure genomic DNA. The quality and quantity of the DNA were confirmed using a Bioanalyzer (Agilent Technologies, Santa Clara, CA, USA) and a Qubit 2.0 Fluorometer (Invitrogen, Life Technologies, CA, USA). Morphological classification and mitochondrial COI markers were used to identify the species [15]. 

DNA (1 µg) was sheared into 350 bp fragments using a Covaris S2 system (Covaris, Woburn, MA, San Diego, CA, USA) in accordance with the manufacturer’s protocol. The *C. dewitti* DNA library was prepared using the Illumina TruSeq DNA PCR-Free Library Prep protocol (Illumina San Diego, CA, USA). The quality check was performed using a Bioanalyzer (Agilent Technologies) and was used to cluster on the Illumina cBOT station and sequence paired ends for 101 cycles using the Illumina Novaseq 6000 platform (Illumina).

### 2.2. Data Analysis, Genome Assembly, and Microsatellite Detection

The quality values of Q20 (percentage of bases whose base call accuracy exceeds 99%) and Q30 (percentage of bases whose base call accuracy exceeds 99.9%) and the GC content were estimated by seqtk version 1.3 (Available on-line: http://github.com/lh3/seqtk, accessed on 22 February 2022) with the primary Illumina paired-end data. Illumina paired-end data were used to estimate the genome size of *C. dewitti* using Jellyfish version 2.1.4 [16]. K-values of 17, 19, and 25 were used, and the 19-mer was completely visualized and the unique sequence, heterozygosity, duplication, and error rates were determined using GenomeScope [17]. The de novo draft genome assembly was conducted using Maryland Super-Read Celera Assembler (MaSuRCA) version 3.3.4 [18], and the contig-level assembly statistics were calculated using the assemblathon_stats.pl script (available online: https://github.com/ucdavis-bioinformatics/assemblathon2-analysis (accessed on 5 March 2022). We checked the assessment of the completeness of the MaSuRCA assembly using Benchmarking Universal Single-Copy Orthologs (BUSCO) version 5.4.2 [19] with the Eukaryota odb 10 database.

The crocodile icefish draft genome assembly was used to identify microsatellite repeat units using the QDD pipeline version 3.1.2 [14]. Using default parameters, three QDD pipeline steps were performed. The three QDD steps that were added were contig 1 (step 1), make_cons 0 (step 2), and contig 1 (step 3). The output of the final step was used to choose the best primer pairs for microsatellite validation. The selected candidate primer pairs were filtered using the following parameters: forward and reverse flanking regions between the simple sequence repeat, primer sequences ≥ 20, maximum primer alignment score of 5 or ≥7 motif repeats, extracting singletons, and high-quality primer design [20].

For the microsatellite validation, the pure genomic DNA was isolated using one specimen with phenol-chloroform method. Randomly selected, 30 primer pairs were used for validation via PCR carried out in a Thermal Cycler Dice Touch (Takara Bio, Shiga, Japan). The PCR tube (50 µL) contained 1 µL genomic DNA (50 ng/µL), 0.5 µL (20 pmol/L) of each forward and reverse primer, 25 µL of 2X EmeraldAmp PCR Master Mix (Takara Bio, Kusatsu, Japan), and 23 µL of double-distilled water. The PCR conditions were as follows: 2 min at 94 °C; 35 cycles at 94 °C for 30 s, 60 °C for 30 s, and 70 °C for 1 min; and a final extension at 72 °C for 10 min. Using the PCR product, three replicates were performed and the final validations were visualized by 4% agarose gel electrophoresis for 30 min at full volt and were displayed using a 20 bp DNA ladder (Takara Bio, Kusatsu, Japan).

## 3. Results and Discussion

### 3.1. Genome Sequencing Data Analysis and Genome Survey

A total of 60.43 Gb of sequencing data was generated using the paired-end library method on the Illumina Novaseq 6000 platform. Q20 and Q30 should be at least 90% and 85%, respectively, for highly accurate sequencing data on the Illumina NGS platform [21]. The Q20 and Q30 values of the primary Illumina paired-end data were 96.1% and 91.0%, respectively, using the seqtk script. The GC content of *C. dewitti* was 49.9% (Table 1), which did not affect the genome assembly.

**Figure 1:**
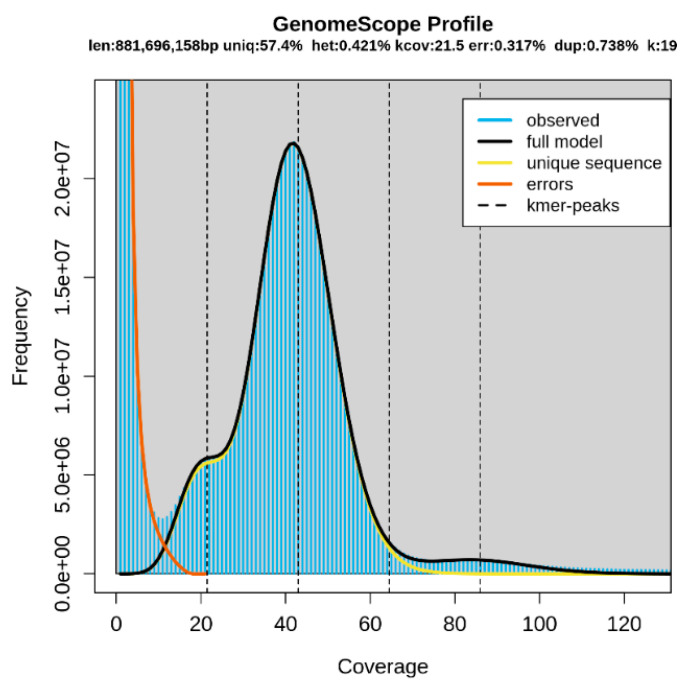
K-mer analysis of *Chionobathyscus dewitti* with GenomeScope (K = 19).

Genome sequencing data (Table 1) were used to predict the genome size via K-mer analysis. K-values of 17, 19, and 25 were used, and the 19-mer was best estimated. Based on the 19-mer distribution, the estimated genome size was 881.7 Mb in total, and the unique sequence, heterozygosity, error, and duplication rates were 57.4%, 0.421%, 0.317%, and 0.738%, respectively (Figure 1). Other estimated K-values used and the genome size, heterozygosity, error, and duplication rates are displayed in Supplementary Table S1.

The graph shows the overall K-mer and genome sizes of *C. dewitti*. Len, estimated total genome length; Uniq, unique portion of the genome (not repetitive); Het, heterozygosity rate; Kcov, K-mer coverage for the heterozygous bases; Err, error rate; Dup, duplication rate. The blue bar in the graph represents the observed K-mer, and the yellow and orange lines in the graph represent the errors and unique sequences, respectively. The statistics of the overall analysis are displayed in the graph. 

### 3.2. Genome Assembly and Microsatellite Detection 

Genome sequencing data of *C. dewitti* were assembled into contigs using MaSuRCA. Using the assemblathon_stats.pl script, we obtained 695,247 scaffolds with a total size of 897,784,561 bp. The longest scaffold was 51,375 bp and the N50 scaffold length was 2401 bp. The GC content of the genome assembly was 41.72% (Table 2). Using BUSCO and the Eurkayota database, we validated assembly contiguity and evaluated the completeness of the assembled genome. Among the 255 total BUSCO groups searched, 112 (43.9%) BUSCO core genes were identified as complete BUSCO profiles. Of these, 110 (43.1%) were single-copy and 2 (0.8%) were duplicated BUSCOs. The fragmented and missing BUSCOs were 115 (45.1%) and 28 (11%), respectively (Table S2). The results of the assembled genome provide preliminary data for whole-genome studies to accomplish accurate assembly and chromosomal-level scaffolding using long-read sequencing and chromosome conformation capture technologies.

**Figure 2:**
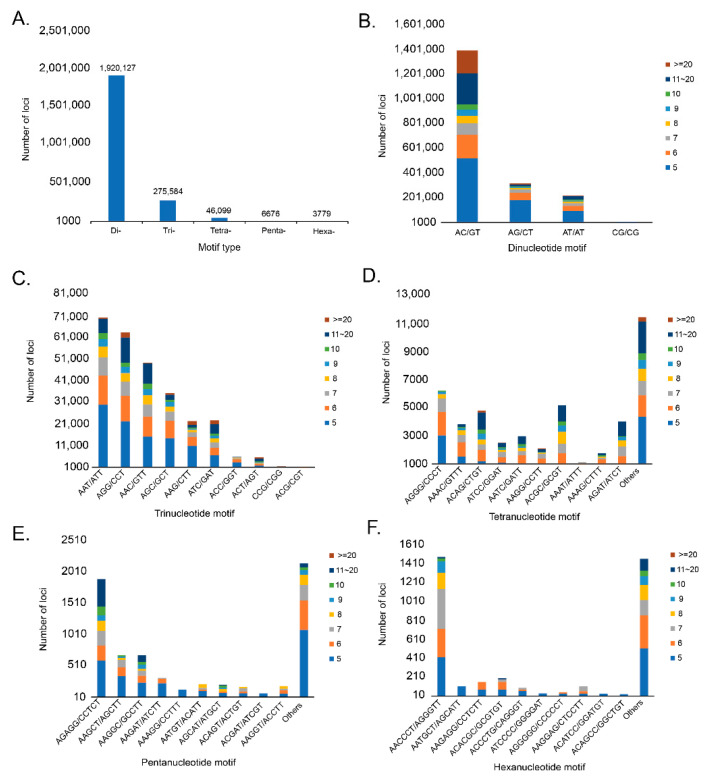
Type and frequency of microsatellite motifs in *Chionobathyscus dewitti* genome. (**A**) represents the frequency of different microsatellite motif types and (**B**) represents the frequency of different dinucleotide microsatellite motifs. (**C**–**F**) represent the frequency of different trinucleotide, tetranucleotide, pentanucleotide, and hexanucleotide microsatellite motifs.

The assembled genome was used to identify microsatellites using QDD version 3.1.2. A total of 2,252,265 microsatellite motifs were identified. Among these, dinucleotide repeats (1,920,127; 85.25%) had the highest rate, followed by trinucleotide repeats (275,584; 12.24%), tetranucleotide repeats (46,099; 2.05%), pentanucleotide repeats (6676; 0.30%), and hexanucleotide repeats (3799; 0.16%; Figure 2). The most frequent motif in dinucleotides was AC/GT (72.23%), followed by AG/CT (16.41%), AT/AT (11.30%), and CG/CG (0.06%). The most frequent motifs in the trinucleotide repeats were AAT/ATT (25.32%), followed by AGG/CCT (22.85%), AAC/GTT (17.76%), AGC/GCT (12.72%), AAG/CTT (8.06%), ATC/GAT (8.17%), AAC/GGT (2.09%), ACT/AGT (2.03%), CCG/CGG (0.53%), and ACG/CGT (0.47%). Among tetranucleotide, pentanucleotide, and hexanucleotide repeats, the most frequent motifs were AGGG/CCCT (13.50%), AGAGG/CCTCT (28.30%), and AACCCT/AGGGTT (39.01%). These results confirm that the high mutation rate is closely related to long mutations, which is consistent with the observation that the repeat frequency decreases with repeat length [22].

**Figure 3:**
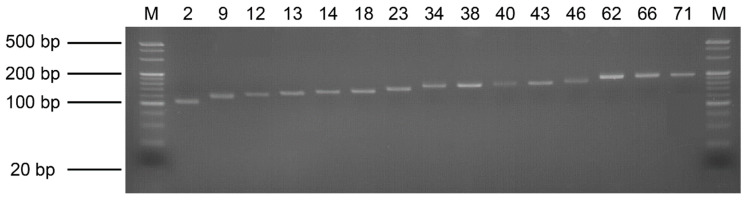
Validation of PCR products representing 15 microsatellite primer pairs. A 4% agarose gel was used for electrophoresis, and M represents the 20 bp DNA marker. The number of the primer pairs are in order.

Using the default parameters of the QDD pipeline and the conditions set forth in [20], we identified 84 primer pairs in the *C. dewitti* draft genome. Thirty primer pairs were randomly selected for verification and used for PCR. As a result, 15 primer pairs were confirmed to yield only a single band, and the primer sizes are shown (Figure 3 and Table 3).

The figure shows the overall type and frequency of microsatellite motifs. The x-axis represents the type of motif, the y-axis represents the number of loci, and the legend displayed on the right represents the number of repeats by different colors. (A) shows different type of motifs in *C. dewitti* genome and the numbers are displayed above the bar chart. (B) shows dinucleotide motif and the repeat motif ‘AC/GT’ was the highest. (C) represents trinucleotide motif and the repeat motif ‘AAT/ATT’ have the highest. (D) represents tetranucleotide motif and among the 10 repeat motifs displayed, ‘AGGG/CCCT’ was the highest, and others were identified as a total of 11,468. (E) shows pentanucleotide motif and among the 10 repeat motifs displayed, ‘AGAGG/CCTCT’ was the highest, and others were identified as a total of 2141. (F) represents hexanucleotide motif and among the 10 repeat motifs displayed, ‘AACCCT/AGGGTT’ was the highest, and others were identified as total of 1453. 

All 15 species of icefish (family Channichthyidae) that live in the Southern Ocean lack hemoglobin [23], and because of the extreme aerobic mode of metabolism, they are thought to have scarce myoglobin, an intracellular respiratory pigment [23]. Both environmental and physiological properties help explain why hemoglobin and myoglobin losses are not fatal at the level of individual organisms. Advance in NGS technology and bioinformatics tools, genome surveys, and K-mer analysis developed rapidly, making these tools efficient in predicting the genome size and characteristics of non-model species [24]. Using flow cytometry, previous studies have estimated that the genome sizes of species living in the Antarctic are approximately 0.7–1.4 Gb [25]. However, with the current advancement of NGS technology, the genome sizes of Antarctic fishes are projected to be 0.6–1 Gb [26,27,28]. The genome size of *C. dewitti* was 0.88 Gb in this study, which was within the range of the previously reported genome sizes of Antarctic fishes. As a result, *C. dewitti*’s genome size is an acceptable outcome. 

The GC content is one of the factors affecting sequence bias and between 30% and 50% does not have a significant effect on genome sequence quality [29,30]. The GC content of the assembled genome was 41.72% (Table 2), which was within the range of 30–50%. The assembled genome provides a preliminary understanding of the genomic characteristics of *C. dewitti*. However, future studies on obtaining high-quality genome assemblies, long-read sequencing technology, and chromosomal-level scaffolding technology are needed.

Microsatellites construct a meaningful distribution of eukaryotic genomes, are highly polymorphic, and have remarkable coding gene sequences in both respects [22]. In the past, it took a lot of time and effort to identify repetitive sequences in genomic DNA to select the appropriate microsatellites [11,12], but now, using the NGS technology, microsatellites can be selected more easily, conveniently, and efficiently [13]. Using assembled draft genome data of *C. dewitti*, 2 252 265 microsatellite motifs were identified, of which dinucleotide repeats (1,920,127; 85.25%) occupied the largest proportion of all repeat motifs. The frequency of di-nucleotide repeats in *C.* *dewitti* species was comparable to that of other fish species evaluated, such as *Pseudosciaena crocea* and *Megalobrama amblycephala* [31,32]. Furthermore, because long mutations are associated with high mutation rates, this result was consistent with data demonstrating that repeat frequency declines with increasing repeat length [22]. This finding was also consistent with previous research on microsatellite repeats in *Danio rerio*, *Oreochromis latipes*, and *Oreochromis niloticus* [33]. The overall tendency of motif frequency showed a pattern similar to that of previous studies on other fish [34]. These results could be relevant in identifying *C. dewitti* microsatellite markers. 

Based on microsatellite motif analysis and select candidate primer pairs according to the parameter of QDD pipeline, we identified 84 primer pairs and randomly selected 30 primer pairs to verify by PCR. Finally, out of the 30 primer pairs, 15 primer pairs were confirmed with a single band. Recent research on the notothenioid fish *Lepidonotothen nudifrons* to evaluate the genetic structure [35] and a study of population genetics in the Patagonian toothfish, *Dissostichus eleginoides* were performed using microsatellite markers [36]. The microsatellite markers that we classified provide a chance to profile the demographic events that occurred in the past and identify the characteristics of local adaptation in genetically different populations [35]. Understanding the importance of these events and the climate challenges facing polar marine organisms is essential for modeling and projecting future population viability for species management and conservation [37].

## 4. Conclusions

The crocodile icefish, *Chionobathyscus dewitti*, belongs to the family Channichthyidae and inhabits the deep sea around the Southern Ocean. The genome of the crocodile icefish was assembled, and microsatellite motifs were identified. The estimated genome size was 0.88 Gb based on K-mer analysis, and a total of 2,252,265 microsatellite motifs were identified. Dinucleotide repeats (1,920,127; 85.25%) had the highest rate. We selected 84 primer pairs based on the assembled genome and 15 primer pairs were validated as microsatellite markers. These genomic data provide the foundation for the development of novel molecular markers in a distinct population. However, more research is needed, such as more accurate genome assembly at the chromosomal level and more microsatellite validation experiments in the future.

## Figures and Tables

**Table 1:** Genome survey sequencing statistics of *Chionobathyscus dewitti*.

Raw Data (bp)	Total Reads	Q20 (%)	Q30 (%)	GC Content (%)
60,426,455,804	400,175,204	96.1	91.0	49.9

**Table 2:** Statistics of *Chionobathyscus dewitti* assembly with MaSuRCA.

	MaSuRCA
Number of scaffolds	695,247
Total size of scaffolds	897,784,561
Longest scaffold	51,375
Number of scaffolds > 1 K nt	222,128 (31.9%)
Number of scaffolds > 10 K nt	6622 (1.0%)
N50 scaffold length	2401
L50 scaffold count	87,813
GC contents (%)	41.72

**Table 3:** 15 microsatellite primer sets of *Chionobathyscus dewitti* used in validation.

Primer No.	Motif	PCR Product Size (bp)	Forward Primer (5′ → 3′)	Tm (°C)	Reverse Primer (5′ → 3′)	Tm (°C)
1	AT 9	105	AACTGCACAGAACAGGGTCA	59.823	AAGCCTCGGTATGCAGAGTG	59.454
2	AC 14	120	GTGAAGAACTTGGTGCTGCC	59.897	CAGGTACGCTAAGCCAGGAG	59.688
3	AC 7	128	AAGTGGGCTGGTGAACTTGT	59.187	TCCCGTGATGTCTTACCGAC	59.741
4	AC 8	133	TAAACCCAGGAGCGTAAGGC	59.824	GCCTCTTGCTCCGACACATA	59.749
5	AC 7	138	CCCTGAATGGCTTCCAACAC	59.964	ACGAGCCTTAATGCACACCA	59.109
6	AC 11	141	AGCATGTGTTCTTGCCAGGA	59.307	CACAGGTGGAGGCTACCTTT	59.889
7	AC 10	149	TGTGTTAAGAGCCGTGCTGT	59.821	CCTCGCTGCCTTTGAGGTAT	59.894
8	AC 17	159	CACCAGGCAGCTCGTTACTA	59.061	GTGTTTACGAACAGCGGGTT	59.469
9	AC 12	161	ACAGCCGGTTGACTGAAACT	59.179	ATCTCCAGTTCTCTGCGTCC	59.82
10	AT 9	163	CATGAGCAATGTTCCGTCGG	59.046	CCTGTTGGAGACACAAAGCC	59.625
11	AC 11	166	CCACAGATGTTGACTTGGCG	59.746	GGCCCGTAACACCCTGTATT	59.481
12	AC 22	171	AGGCATTTAACCTCGGCACA	59.451	GGCTCCTATTTCACCCAGCT	59.962
13	AC 12	190	GAAAGCAGGCACTCAGATGC	59.753	TCATTCCAGCACACTCTCCG	59.549
14	AC 14	192	GTGCATCTTTCTACCGCTGC	59.537	CTGCTCGACCCTGATGACAT	59.625
15	AC 9	194	CGGGTAAACGCTATGGAGGT	59.751	CGGACTCCATACTGTTGGCA	59.537

## Data Availability

The raw data generated by the Illumina Novaseq 6000 platform are displayed and available at NCBI (https://www.ncbi.nlm.nih.gov/bioproject/PRJNA730390 (accessed on 28 August 2022)). The accession number for the BioProject is PRJNA730390 and for the SRA data is SRR14562209.

## Supplementary Materials

The following supporting information can be downloaded at: https://www.mdpi.com/article/10.3390/ani12192598/s1, Table S1: Genome estimation of *Chionobathyscus dewitti* using K-mer analysis; Table S2: Completeness of *Chionobathyscus dewitti* genome assembly estimated by BUSCO.
