# Supplementary material for: Whole-Genome Survey and Microsatellite Marker Detection of Antarctic Crocodile Icefish, Chionobathyscus dewitti
Source: Animals (Basel). 2022 Sep 28;12(19):2598. doi: 10.3390/ani12192598 (PMC9558526; doi:10.3390/ani12192598)
Supplement: Supplementary file 1 [file animals-12-02598-s001.zip › animals-1913590-supplementary.pdf]

Supplementary Table S1. Genome estimation of *Chionobathyscus dewitti* using K-mer anaysis.

K-mer	Genome size (bp)	Heterozygosity (%)	Error (%)	Duplication (%)
17	862,611,855	0.397	0.266	0.787
19	881,696,158	0.421	0.317	0.738
25	925,384,674	0.412	0.313	0.724

Supplementary Table S2. Completeness of *Chionobathyscus dewitti* genome assembly estimated by using BUSCO (Benchmarking Universal Single-Copy Orthologs).

Eukaryote odb10	No.	%
Complete BUSCOs (C)	112	43.9
Complete and single-copy BUSCOs (S)	110	43.1
Complete and duplicated BUSCOs (D)	2	0.8
Fragmented BUSCOs (F)	115	45.1
Missing BUSCOs (M)	28	11.0
Total BUSCO groups searched	255	
